# Real Time Hemodynamic Monitoring During M‐TEER Using Electrical Cardiometry

**DOI:** 10.1002/ccd.31527

**Published:** 2025-04-10

**Authors:** Andreas Goldschmied, Manuel Sigle, Ioannis Toskas, Monika Zdanyte, Mareike Bladt, Meinrad Gawaz, Tobias Geisler

**Affiliations:** ^1^ Department of Cardiology University Hospital Tübingen Tübingen Germany

**Keywords:** electrical cardiometry, hemodynamics, M‐TEER, mitral regurgitation, right heart catheter

## Abstract

**Background:**

Mitral regurgitation is a common valvular dysfunction causing patient morbidity and mortality. Mitral transcatheter edge‐to‐edge repair (M‐TEER) allows grasping of valvular leaflets and approximation via a small implant, thus reducing mitral regurgitation (MR). The implant allows staged leaflet capture and leaflet optimization before it is finally released. Real time hemodynamic monitoring could facilitate procedural success and improve patient outcomes.

**Methods:**

Fourteen patients scheduled for elective M‐TEER were included in this study. Right heart catheterization (RHC) and determination of cardiac stroke volume (SV) using the established Fick method, pulmonary capillary wedge pressure (PCWP), PCWP v‐wave, left atrial (LA) pressure and LA v‐wave were carried out pre and postprocedurally. Concomitantly, real time SV was measured via electrical cardiometry and acquired data were compared.

**Results:**

A significant increase in cardiac stroke volume measured via RHC was observed after successful M‐TEER. Even though pre‐ and postprocedural RHC and electrical cardiometry measurements correlated significantly, electrical cardiometry was not able to reproduce the absolute increase in SV seen on RHC measurements. Furthermore, a decrease in PCWP mean pressure, PCWP v‐wave, LA mean pressure and LA v‐wave, were observed.

**Conclusion:**

Cardiac SV increases after successful M‐TEER as measured via RHC but electrical cardiometry was not able to reproduce these changes in a real time, beat‐to‐beat measurement.

**List of Abbreviations**
3D EROA: three‐dimensional effective regurgitant orifice area
ACE: angiotensin converting enzyme
ARB: angiotensin receptor blocker
ARNI: angiotensin receptor/neprilysin inhibitor
ASA: acetylsalicylic acid
BMI: body mass index
DOAC: direct oral anticoagulant
LA: left atrium
LVEF: left ventricular ejection fraction
MR: mitral regurgitation
MRA: mineralocorticoid receptor antagonist
M‐TEER: mitral transcatheter edge‐to‐edge repair
NYHA: New York Heart Association
OMT: optimal medical therapy
PA mean: mean pulmonary artery pressure
PCWP: pulmonary capillary wedge pressure
RHC: right heart catheter
SGLT2: sodium/glucose cotransporter 2
SV: stroke volume
TOE: transesophageal echocardiography
TR: tricuspid regurgitation

## 1 Introduction

Mitral regurgitation (MR) is the second most common valvular dysfunction in Europe and a substantial contributor to morbidity and mortality [1, 2, 3].

In severe MR, backwards blood flow and increased filling pressures causes pulmonary vein congestions and leads to pulmonary edema as well as postcapillary pulmonary hypertension. Increased right ventricular afterload can in turn cause right ventricular failure and tricuspid regurgitation (TR) which increases central venous pressure. Especially in the presence of low effective stroke volume (SV) due to backwards flow, tissue perfusion is impaired which ultimately causes end organ failure [4].

Mitral transcatheter edge‐to‐edge repair (M‐TEER) is a well‐established minimal invasive procedure to treat MR. Multiple studies have demonstrated reduction in all‐cause mortality, hospitalization for heart failure and noninferiority to surgery in selected patients suffering from secondary MR. The procedure is also recommended in primary MR with high surgical risk [5, 6, 7, 8, 9].

Postinterventional effects on patient hemodynamic parameters like increased SV, increased cardiac output and reduction in systemic vascular resistance have been reported using noninvasive bioimpedance monitoring as well as echocardiography [10, 11].

After successful leaflet grasping, the implant is closed and MR reduced. This can be verified in real time using TOE. If the result is not satisfactory, the implant can be re‐opened and leaflet optimization can be carried out to ensure effective MR reduction. In the final step, the device is released from the catheter.

Electrical cardiometry (ICON®, OSYPKA MEDICAL, Berlin, Germany) is a noninvasive method to measure real time hemodynamic parameters like cardiac SV. The validity of this method has been shown in multiple studies and several settings [12, 13, 14].

In our study, we implemented real time hemodynamic monitoring using the ICON system in patients undergoing M‐TEER and compared cardiac SV to invasive measurements obtained by right heart catheter (RHC) before and after the intervention using the Fick method. The goal is to provide real time feedback on hemodynamic changes after leaflet grasping and approximation. This could help to quantify reduction of MR additionally to echocardiographic imaging and visualize hemodynamic changes to provide better interventional results and improve patient outcome.

## 2 Materials and Methods

### 2.1 Study Design

This single center prospective clinical study was carried out between June and December 2024 at the Department of Cardiology of the University Clinic Tuebingen, Germany. Fourteen consecutive patients who were scheduled for elective M‐TEER using the PASCAL device (Edwards Lifescienc, Irvine, USA) were included. All patients received optimal medical therapy (OMT) before the intervention and were treated according to current guidelines [2]. Inclusion criteria included age> 18years, signed written informed consent and severe MR. Clinical data like age, sex, height and weight as well as data on prior medical history, current medication and laboratory values were collected from the patient chart. The study was approved by the institutional ethics committee (238/2018BO2) and complies with the declaration of Helsinki and the good clinical practice guidelines [15, 16, 17].

### 2.2 Electrical Cardiometry

Electrical cardiometry using the ICON system (OSYPKA MEDICAL, Berlin, Germany) was established before the start of the intervention. The four electrodes were placed on the patients’ neck and thorax according to the manufacturer's instructions. This way, blood flow along the ascending and descending aorta is measured, thus estimating forward stroke volume. Patient data like height, weight, age, sex, hemoglobin levels and current oxygen saturation were inputted into the device. Events like RHC measurements and PASCAL placement were marked using timestamps provided by the manufacturer's software. The data which included minute‐by‐minute measurements were exported into Microsoft Excel (Microsoft Corporation, Redmond, USA). SV at the time of RHC was calculated as an average across 5 min (five measurements).

### 2.3 Invasive Hemodynamic Readings

RHC was carried out immediately before the start and after the end of the M‐TEER procedure. Under fluoroscopic guidance, a balloon catheter was inserted through the femoral vein into the right atrium, the right ventricle, the pulmonary artery and into a pulmonary capillary wedge position. Pressure readings were taken in all four positions. Additionally, pressure readings of the left atrium (LA) before and after PASCAL placement were taken through the M‐TEER guiding catheter. To calculate SV using the established Fick method, mixed venous oxygen saturation was measured in the pulmonary artery. Simultaneously, arterial oxygen saturation was measured by drawing blood from an arterial line established in the radial artery for invasive blood pressure monitoring during deep sedation. Arteriovenous oxygen difference before and after M‐TEER are demonstrated in Supporting Information S1: Table 1.

### 2.4 Study Objectives

The primary endpoint was defined as correlation between SV measurements using the Fick method (gold standard) and simultaneous ICON measurement before and after M‐TEER as well as change in SV. Secondary endpoints include change in hemodynamic parameters like SV, mean pulmonary artery pressure (PA mean), mean pulmonary capillary wedge pressure (PCWP), pulmonary capillary wedge pressure v‐wave, mean left atrial (LA) pressure, LA v‐wave and echocardiographic three‐dimensional effective regurgitant orifice area (3D EROA).

### 2.5 Statistical Analysis

All statistical analysis was performed using IBM SPSS Statistics Version 28.0 (IBM, Armonk, USA). Data were tested for normal distribution using the Kolmogorov–Smirnov test. Continuous variables were displayed as mean ± standard deviation and categorial variables were displayed as counts and percentages. Paired *t*‐tests or Wilcoxon signed‐rank tests were used to compare hemodynamic and echocardiographic data before and after successful M‐TEER. Pearson's correlation coefficient was calculated to compare measurements on SV obtained by ICON and RHC. A two‐sided alpha level of < 0.05 was considered statistically significant for all tests.

### 2.6 Visualization

IBM SPSS Statistics Version 28.0 (IBM, Armonk, USA), Microsoft Power Point (Microsoft Corporation, Redmond, USA) and RStudio (Posit PBC, Boston, USA) package “ggplot2” were used to generate figures.

## 3 Results

### 3.1 Baseline Characteristics

The mean age of the overall cohort was 78.8 ± 4.5 years and 71% of participants were male. The majority of patients (57%) was classified as NYHA III heart failure and mean left ventricular ejection fraction (LVEF) was 39.6 ± 15.1%. Most individuals suffered from secondary MR (71.4%) and were treated with a one (50%) or two (42.9%) device strategy. The full baseline characteristics are demonstrated in Table 1.

**TABLE 1:** Patient characteristics of study population (*n* = 14).

Variable	Value
Age (years)	78.8 ( ± 4.5)
Gender (male)	10 (71.4)
BMI (kg/m²)	26.3 ( ± 3.1)
ß‐blocker	14 (100)
ACE inhibitor	3 (21.4)
ARB	3 (21.4)
ARNI	5 (35.7)
MRA	9 (64.3)
DOAC	11 (78.6)
SGLT2 inhibitor	13 (92.9)
ASA	4 (28.6)
Platelet aggregation inhibitor	3 (21.4)
LVEF (%)	39.6 ( ± 15.1)
NYHA classification II	5 (35.7)
NYHA classification III	8 (57.1)
NYHA classification IV	1 (7.1)
One device implanted	7 (50)
Two devices implanted	6 (42.9)
Three devices implanted	1 (7.1)
Primary MR	3 (21.4)
Secondary MR	10 (71.4)
Mixed MR	1 (7.1)
Arterial hypertension	7 (50)
Diabetes mellitus	4 (28.6)
Coronary artery disease	7 (50)
Atrial fibrillation	11 (78.6)
Chronic kidney disease	5 (35.7)

*Note:* Continuous variables are displayed as mean ± standard deviation and categorial variables are displayed as counts and percentages.

Abbreviations: ACE, angiotensin converting enzyme; ARB, angiotensin receptor blocker; ARNI, angiotensin receptor/neprilysin inhibitor; ASA, acetylsalicylic acid; BMI, body mass index; DOAC, direct oral anticoagulant; LVEF, left ventricular ejection fraction; MRA, mineralocorticoid receptor antagonist; MR, mitral regurgitation; NYHA, New York Heart Association; SGLT2, sodium/glucose cotransporter 2.

### 3.2 Stroke Volume Increases After M‐TEER

**FIGURE 1:**
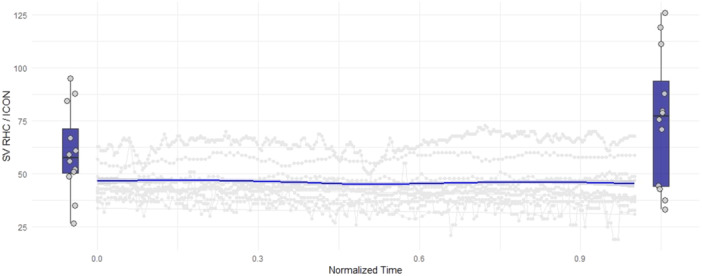
Hemodynamic changes during M‐TEER. Normalized procedural time is displayed on the x‐axis while stroke volume is displayed on the y‐axis. Boxplots indicate stroke volume measurements via right heart catheter, gray lines show individual measurements via ICON during the procedure. The blue line depicts average stroke volume of all 14 patients measured via ICON. [Color figure can be viewed at wileyonlinelibrary.com]

**FIGURE 2:**
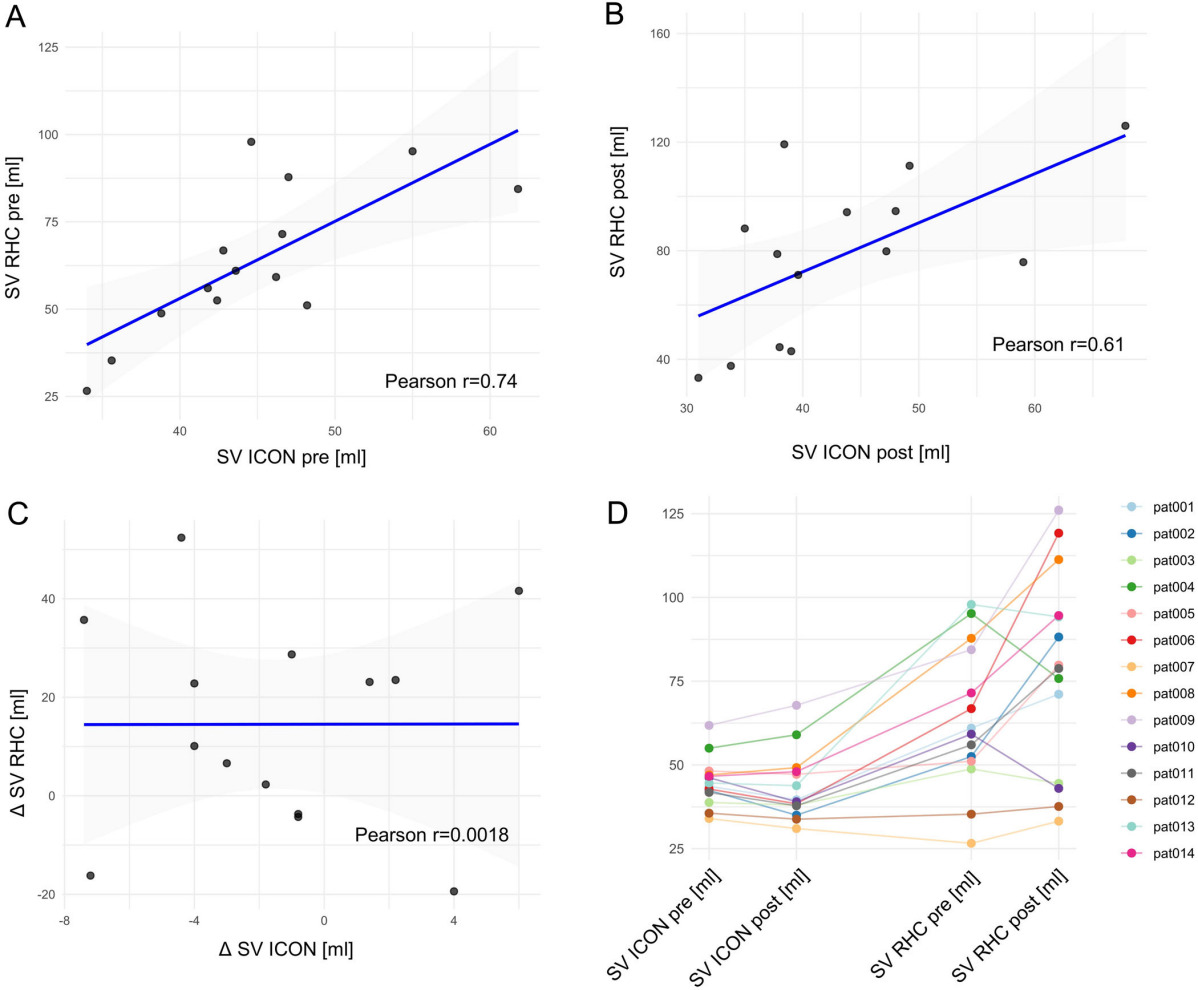
Correlation between stroke volume measured via right heart catheter and ICON. Scatter plots demonstrating correlation between the two methods pre M‐TEER (A), post M‐TEER (B) and change in stroke volume (C). Stroke volume in mL measured via ICON is displayed on the x‐axis while stroke volume measured via right heart catheter in mL is displayed on the y‐axis. Individual patient data on stroke volume measured via the two methods before and after M‐TEER (D). Stroke volume in mL is displayed on the y‐axis while measuring method is demonstrated on the x‐axis. [Color figure can be viewed at wileyonlinelibrary.com]

Mean SV as measured via RHC increased significantly after successful M‐TEER (Table 2). Boxplots showing SV pre and post M‐TEER are demonstrated in Figure 1. Assessment of SV pre M‐TEER correlated significantly between RHC and ICON (Figure 2A/Table 2). However, the increase in SV measured by RHC could not be detected using the ICON method (Figure 1). Even though absolute SV measured via RHC and ICON did still significantly correlate post M‐TEER (Figure 2B/Table 2), change in SV (delta SV) did not (Figure 2C/Table 2). Overall SV measured via ICON was lower than measurements via RHC (Figure 1). Individual patient data on stroke volume measured via the two methods before and after M‐TEER are demonstrated in Figure 2D.

**TABLE 2:** Results of statistical testing.

Variable	Test	Figure	*p*‐value
Delta stroke volume RHC pre versus post M‐TEER	Paired *t*‐test	2	**0.026**
Stroke volume pre M‐TEER ICON versus RHC	Pearson's correlation coefficient	3	**0.003**
Stroke volume post M‐TEER ICON versus RHC	Pearson's correlation coefficient	3	**0.021**
Delta stroke volume ICON versus RHC	Pearson's correlation coefficient	3	0.995
PCWP mean pre versus post M‐TEER	Paired *t*‐test	4	0.355
PCWP v‐wave pre versus post M‐TEER	Wilcoxon signed‐rank tests	4	0.151
LA mean pressure pre versus post M‐TEER	Wilcoxon signed‐rank tests	4	0.917
LA v‐wave pre versus post M‐TEER	Wilcoxon signed‐rank tests	4	**0.049**
PA mean pressure pre versus post M‐TEER	Wilcoxon signed‐rank test	4	0.556
3D EROA pre versus post M‐TEER	Paired *t*‐test	4	**< 0.001**
Delta 3D EROA versus delta stroke volume RHC	Pearson's correlation coefficient	S1	0.420

*Note:* Significant *p*‐values of test results are displayed in bold.

Abbreviations: 3D EROA, three‐dimensional effective regurgitant orifice area; LA, left atrium; M‐TEER, mitral transcatheter edge‐to‐edge repair; PCWP, pulmonary capillary wedge pressure; PA, pulmonary artery; PA, RHC, right heart catheter.

### 3.3 Hemodynamic and Echocardiographic Parameters Change After M‐TEER

**FIGURE 3:**
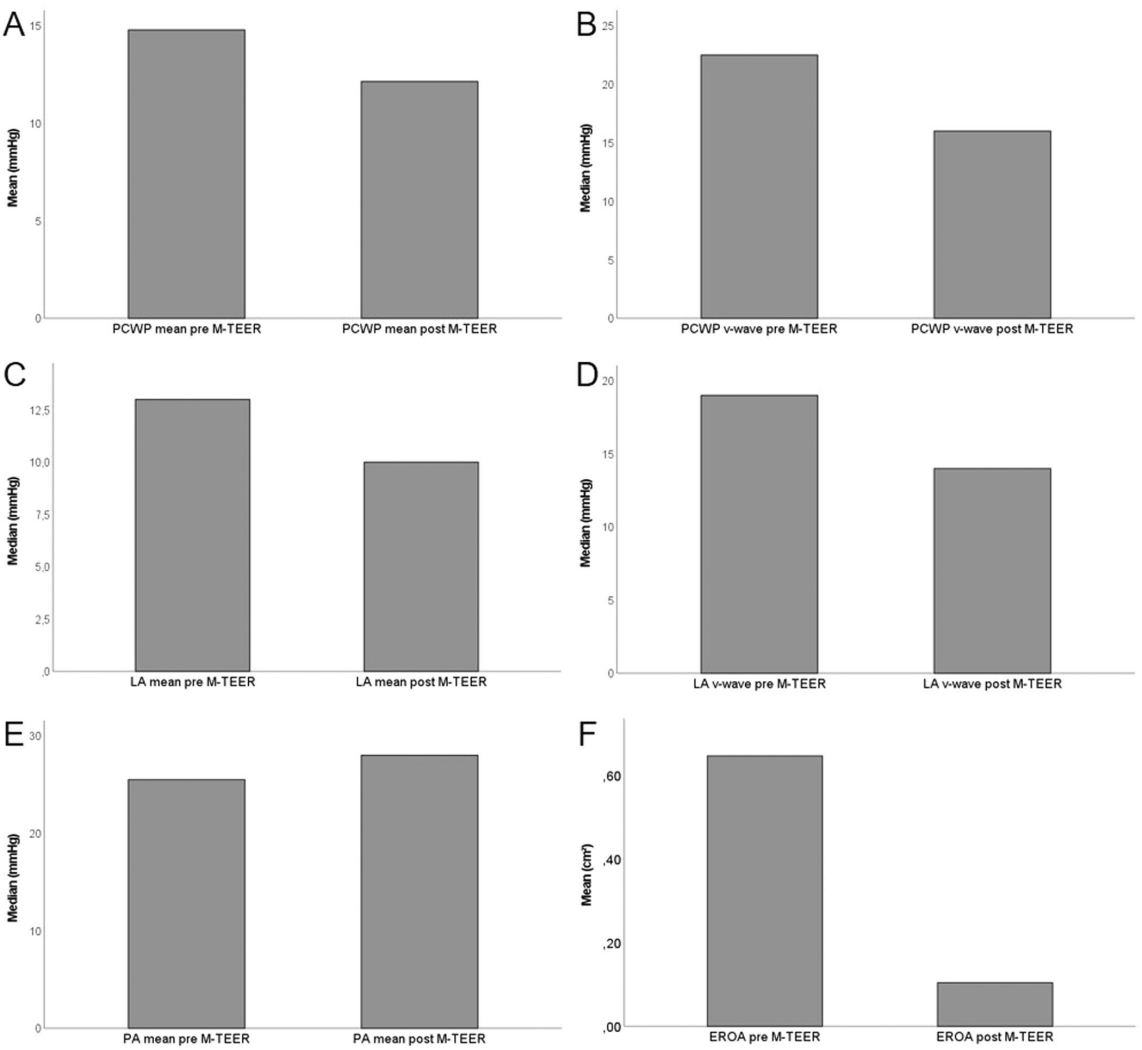
Bar charts of hemodynamic and echocardiographic parameters pre and post M‐TEER. Bar charts indicating change of PCWP (A), PCWP v‐wave (B), LA pressure (C), LA v‐wave (D), PA mean pressure (E) and 3D EROA (F). The measured parameter is displayed on the x‐axis while means for normally distributed data and the median for non‐normally distributed data is shown on the y‐axis.

Hemodynamic parameters obtained via RHC changed after successful M‐TEER. Mean PCWP (Figure 3A), PCWP v‐wave (Figure 3B), mean LA pressure (Figure 3C) and LA v‐wave (Figure 3D) all decreased whereas mean PA pressure increased slightly (Figure 3E). Likely due to the relatively small sample size, only the decrease in LA v‐wave was statistically significant (Table 2). To investigate echocardiographic success of the procedure, 3D EROA measurements were carried out pre and postinterventionally. As shown in Figure 3F, D EROA was reduced dramatically after successfully M‐TEER. This reduction reached statistical significance (Table 2). Interestingly, reduction of EROA did not correlate with increase in SV as measured by RHC (Table 2, Supporting Information S1: Figure 1).

## 4 Discussion

In the present study we were able to demonstrate that M‐TEER has immediate hemodynamic effects in patients presenting with severe MR. Invasively measured SV increased significantly while LA mean pressure, LA v‐wave, PCWP mean pressure and PCWP v‐wave were all lower in the postprocedural measurement.

Even though significant correlation between initial measurements and postinterventional measurements could be observed, electrical cardiometry appeared to underestimate SV when compared to the gold standard method in this patient collective. However, in our specific setting, reliable readings of relative changes for periinterventional monitoring of “hemodynamic success” would be sufficient.

Unfortunately, electrical cardiometry was not able to detect the increase in SV in the real time, beat‐to‐beat measurement. The observed increase in SV via the gold standard method was from a mean of 64 mL/beat to 78 mL/beat (18%). While statistically significant, this change might be too subtle to be picked up by electrical cardiometry. However, median signal quality readings provided by the ICON device were at 90% which should be sufficient.

As mentioned above, PCWP mean pressure, PCWP v‐wave, LA mean pressure and LA v‐wave all decreased after successful M‐TEER. Only the increase in LA v‐wave reached statistical significance. However, this is likely to be due to the relatively small sample size as all four parameters show a clear trend. Apparently, pulmonary congestions caused by backwards flow secondary to severe MR is immediately relieved after M‐TEER in our collective.

In this study, we used 3D EROA as a parameter for echocardiographic success of the M‐TEER procedure. Our results show a reduction of mean 3D EROA of 0.65 cm^2^ to 0.1 cm^2^. This not only demonstrated a significant absolute (0.55 cm^2^) and relative (85%) reduction in EROA but also emphasizes that our collective suffered from very severe MR at the start of the procedure.

Interestingly, the reduction in MR as measured via 3D EROA did not correlate with the increase in SV as measured via RHC (Supporting Information S1: Figure 1). This demonstrates that multiple factors likely play a role in SV changes after M‐TEER. Simard et al. were able to show that reduction in mean LA pressure and LA v‐wave depends on MR pathophysiology and Markus et al. demonstrated that particularly patients with poor kidney function showed improvement in cardiac output as measured via noninvasive methods after successful M‐TEER [11, 18]. This highlights the necessity for a multimodal approach when assessing periprocedural success of M‐TEER.

To our knowledge, this is the first study attempting to monitor real time hemodynamic changes during M‐TEER. Since both available systems allow for release and re‐grasping of mitral valve leaflets, real time hemodynamic monitoring would enable to observe changes of cardiac SV before device release. Unfortunately, electrical cardiometry using the ICON system was not able to pick up changes in SV measured via RHC. However, different systems or software updates could enable hemodynamic guidance during M‐TEER which might improves patient outcome.

## 5 Limitations

Since oxygen consumption was assumed and not actually measured, measurement inaccuracies with the RHC (Fick) method cannot be ruled out. Furthermore, we cannot exclude fluctuation in patient oxygen consumption during deep sedation which could influence calculation of stroke volume. Also, the sample size was relatively small to investigate the secondary endpoints. This is likely the reason why most secondary endpoints did not reach statistical significance despite clear trends.

## 6 Conclusion

SV as measured via RHC (Fick method) increases significantly after successful M‐TEER. Unfortunately, this change was not captured via electrical cardiometry making this method currently unsuitable for beat‐to‐beat monitoring during M‐TEER. Furthermore, a decrease in PCWP mean, PCWP v‐wave, LA mean pressure and LA v‐wave could be observed.

## Ethics Statement

The study complies with the declaration of Helsinki and good clinical practice guidelines and was approved by the responsible local ethics committee (project number 238/2018BO2).

## Consent

Written informed consent was obtained in all patients participating in the study.

## Conflicts of Interest

The authors declare no conflicts of interest.

## Supporting information


**Supporting figure 1:** Correlation between change in stroke volume measured via right heart catheter and change in 3D EROA. Scatter plot demonstrating correlation between delta stroke volume and delta 3D EROA **(A)**. Change in echocardiographic 3D EROA in cm^2^ is demonstrated on the x‐axis while change in stroke volume in ml measured via right heart catheter is demonstrated on the y‐axis. Individual patient data on change in 3D EROA after M‐TEER **(B)**. Timing of measurement and change in 3D EROA is demonstrated on the x‐axis while 3D EROA in cm^2^ is given on the y‐axis.

**Supporting table 1:** Arteriovenous O2 difference before and after successful M‐TEER. A‐V – arteriovenous, M‐TEER ‐ Mitral Transcatheter Edge‐to‐Edge Repair.

## Data Availability

Study data are available from the corresponding author upon reasonable request.
