# Supplementary figures and images for: Real Time Hemodynamic Monitoring During M‐TEER Using Electrical Cardiometry
Source: Catheter Cardiovasc Interv. 2025 Apr 10;106(1):196–202. doi: 10.1002/ccd.31527 (PMC12231150; doi:10.1002/ccd.31527)

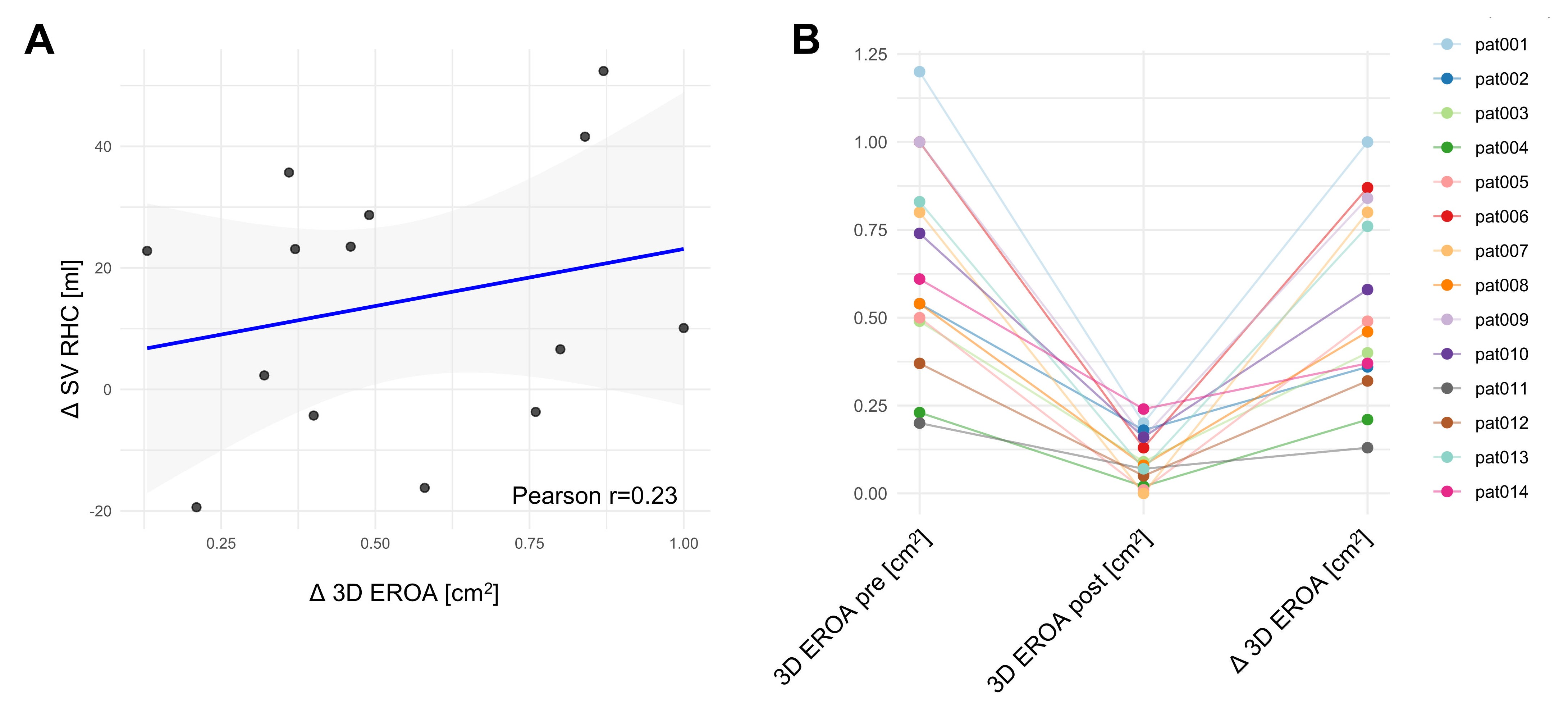

Supplement: Supplementary file 1 — Supporting figure 1: Correlation between change in stroke volume measured via right heart catheter and change in 3D EROA. Scatter plot demonstrating correlation between delta stroke volume and delta 3D EROA (A). Change in echocardiographic 3D EROA in cm2 is demonstrated on the x‐axis while change in stroke volume in ml measured via right heart catheter is demonstrated on the y‐axis. Individual patient data on change in 3D EROA after M‐TEER (B). Timing of measurement and change in 3D EROA is demonstrated on the x‐axis while 3D EROA in cm2 is given on the y‐axis. [file CCD-106-196-s002.tiff]
