# Supplementary material for: Real Time Hemodynamic Monitoring During M‐TEER Using Electrical Cardiometry
Source: Catheter Cardiovasc Interv. 2025 Apr 10;106(1):196–202. doi: 10.1002/ccd.31527 (PMC12231150; doi:10.1002/ccd.31527)
Supplement: Supplementary file 2 — Supporting table 1: Arteriovenous O2 difference before and after successful M‐TEER. A‐V – arteriovenous, M‐TEER ‐ Mitral Transcatheter Edge‐to‐Edge Repair. [file CCD-106-196-s001.docx]

**Supplementary table 1: Arteriovenous O^2^ difference before and after successful M-TEER**

| **Patient** | **A-V O2 difference pre M-TEER (ml/l)** | **A-V O2 difference post M-TEER (ml/l)** |
| --- | --- | --- |
| 1 | 54.07 | 51.28 |
| 2 | 63.39 | 36.04 |
| 3 | 57.81 | 59.38 |
| 4 | 49.50 | 48.84 |
| 5 | 52.23 | 43.33 |
| 6 | 50.17 | 29.87 |
| 7 | 120.77 | 99.55 |
| 8 | 39.44 | 33.78 |
| 9 | 35.98 | 31.28 |
| 10 | 49.02 | 58.26 |
| 11 | 54.43 | 45.67 |
| 12 | 89.68 | 89.97 |
| 13 | 40.80 | 46.58 |
| 14 | 51.79 | 37.86 |

A-V – arteriovenous, M-TEER - Mitral Transcatheter Edge-to-Edge Repair
